# Diphenhydramine, Sodium Bicarbonate, or Combination for Acute Peripheral Vertigo: A Randomized Clinical Trial

**DOI:** 10.1001/jamanetworkopen.2025.41472

**Published:** 2025-11-06

**Authors:** Chien-Yu Chi, Yun-Chang Chen, Ming-Tai Cheng, Kai-Chieh Chang, Yen-Pin Chen, Min-Shan Tsai, Wei-Tien Chang, Matthew Huei-Ming Ma, Chien-Hua Huang, Wen-Chu Chiang

**Affiliations:** 1 Department of Emergency Medicine, National Taiwan University Hospital Yunlin Branch, Douliu, Taiwan; 2 Graduate Institute of Clinical Medicine, National Taiwan University College of Medicine, Taipei, Taiwan; 3 Department of Emergency Medicine, National Taiwan University Hospital and College of Medicine, Taipei, Taiwan; 4 Department of Neurology, National Taiwan University Hospital Yunlin Branch, Douliu, Taiwan

## Abstract

**Importance:**

Intravenous diphenhydramine is commonly used to treat acute peripheral vertigo but may cause sedation. Sodium bicarbonate, an alternative therapy used in Eastern countries, may relieve vertigo symptoms without lethargy. However, comparative evidence is limited.

**Objective:**

To evaluate the efficacy and safety of diphenhydramine, sodium bicarbonate, and their combination in treating acute peripheral vertigo among emergency department (ED) patients.

**Design, Setting, and Participants:**

A triple-arm, double-blind, randomized clinical trial was conducted at the National Taiwan University Hospital, Yunlin Branch ED, from January 17 to November 14, 2023. Adults with peripheral vertigo within 24 hours of onset were enrolled. Exclusion criteria included refusal, pregnancy, prior antivertigo medication use, symptom duration of more than 24 hours, heart failure, chronic kidney disease, or drug allergy.

**Interventions:**

Patients were randomized in a 1:1:1 ratio to receive 30 mg of intravenous diphenhydramine (group A), 66.4 mEq (approximately 1 mEq/kg) of sodium bicarbonate (group B), or both agents (group C).

**Main Outcomes and Measures:**

The primary outcome was change in vertigo severity using a 10-point visual analog scale (VAS) at 60 minutes after treatment. Key secondary outcomes included change in nausea severity using a 10-point VAS, rescue medication use, ED length of stay, and adverse effects. Analysis was performed on a modified intention-to-treat basis.

**Results:**

Among 222 patients (mean [SD] age, 57.9 [17.6] years; 150 women [67.6%]), group C had greater improvement in vertigo severity than group A (mean [SD] VAS score, –5.6 [2.1] vs –4.4 [2.7]; *P* = .01), with the treatment effect most pronounced among younger male patients without a history of vertigo. Group B showed similar VAS improvement compared with group A (mean [SD] VAS score, –5.1 [2.2] vs –4.4 [2.7]; *P* = .34). Rescue therapy was used less frequently in group C (17.8% [13 of 73]) than in group A (46.7% [35 of 75]; *P* < .001). No differences were observed between groups in nausea scores or ED length of stay. Lethargy was more common in diphenhydramine groups than in the sodium bicarbonate group (moderate lethargy: group A, 38.7% [29 of 75] and group C, 30.1% [22 of 73] vs group B, 8.1% [6 of 74]; *P* < .001). Injection discomfort was more common with sodium bicarbonate (group A, 8.0% [6 of 75]; group B, 17.6% [13 of 74]; group C, 28.8% [21 of 73]; *P* = .004).

**Conclusions and Relevance:**

In this randomized clinical trial of patients with acute peripheral vertigo, combination therapy provided better symptom relief than diphenhydramine alone. Further studies should focus on the determination of peripheral vertigo types that can benefit from sodium bicarbonate and the underlying mechanisms.

**Trial Registration:**

ClinicalTrials.gov Identifier: NCT05676216

## Key Points

**Question:**

Does intravenous sodium bicarbonate alone or with diphenhydramine provide better symptom relief than diphenhydramine alone for adults with acute peripheral vertigo?

**Findings:**

In this randomized clinical trial of 225 patients, combination therapy with diphenhydramine and sodium bicarbonate produced greater improvement in vertigo severity at 60 minutes and reduced the need for rescue medication compared with diphenhydramine alone. Sedation occurred more often with diphenhydramine, whereas sodium bicarbonate caused more injection discomfort.

**Meaning:**

This study suggests that combination therapy with diphenhydramine and sodium bicarbonate may offer more effective relief of acute peripheral vertigo symptoms than diphenhydramine alone and could reduce the need for additional treatment.

## Teaser

This randomized clinical trial evaluates the efficacy and safety of intravenous sodium bicarbonate, diphenhydramine, and their combination in treating acute peripheral vertigo among emergency department patients.

## Introduction

Vertigo is a spinning sensation often accompanied by nausea and vomiting. In Taiwan, approximately 3.1% of adults are affected by vertigo, with approximately 37% experiencing recurrence within 1 year.^1^ The sensation of disequilibrium, along with severe nausea and vomiting, often compels patients with vertigo to seek emergency department (ED) assistance.

In the ED, the primary task in the management of acute vertigo is differentiating life-threatening central vertigo.^2,3,4^ Intravenous injection therapy is often used to quickly alleviate peripheral vertigo symptoms.^5,6^ A recent meta-analysis reported that antihistamines, compared with benzodiazepines, significantly improved vertigo symptoms while demonstrating comparable effectiveness with other treatment regimens.^7^ However, approximately 15% to 26% of patients experience fatigue after intravenous administration of antihistamines.^8,9,10^ This fatigue may prolong the observation period in the ED. In addition, there is an increased fall risk after treatment with antihistamines, particularly among older individuals.^11^ Therefore, several studies aimed to investigate more suitable treatment approaches for patients with acute vertigo.^8,9,10,12,13,14,15^

Sodium bicarbonate has traditionally been used to treat acute hyperkalemia, acidosis, and blood and urine alkalinization.^16,17^ There have been reports of sodium bicarbonate use in the treatment of dizziness and vertigo in Taiwan and Japan.^18,19^ Possible mechanisms include increased inner ear perfusion and medial vestibular nucleus neural inhibition.^20,21,22,23^ Unlike antihistamines and benzodiazepines, sodium bicarbonate does not cause drowsiness, fatigue, or other severe adverse effects. However, to date, no standardized comparison report has evaluated the efficacy of sodium bicarbonate in the treatment of peripheral vertigo.

This study aimed to evaluate the efficacy of sodium bicarbonate alone and in combination with an antihistamine compared with an antihistamine alone as treatment for acute peripheral vertigo in patients visiting the ED. We considered that these 2 treatments have different mechanisms of action; therefore, we hypothesized that the 3 treatment approaches would exhibit different efficacies.

## Methods

### Trial Design and Setting

This study was conducted as a 1:1:1 triple-arm, double-blinded randomized clinical trial, in which patients and outcome assessors were blinded to treatment allocation. It was conducted at the National Taiwan University Hospital’s ED in Yunlin, Taiwan, from January 17 to November 14, 2023. The trial protocol is available in Supplement 1. The hospital serves approximately 300 000 people, has approximately 1000 beds, and receives approximately 3500 ED visits each month. Emergency specialist physicians provide emergency services. Under normal circumstances, all patients in the ED can be treated within 30 minutes, with immediate care provided to those presenting with life-threatening conditions. In our setting, the median time from ED triage to discharge for patients with peripheral vertigo is approximately 2 hours, which includes triage, physician evaluation, medication administration, and observation for symptom improvement. This investigator-initiated trial was approved by the National Taiwan University Research Committee. Patients provided written informed consent. The trial protocol was registered and published at ClinicalTrials.gov (NCT05676216). The report adhered to the Consolidated Standards of Reporting Trials (CONSORT) reporting guideline.

### Participants

Patients aged 18 years or older who experienced vertigo onset within the past 24 hours were included in this trial. Exclusion criteria included pregnancy, 5 vertigo episodes over 24 hours, antivertigo medication use after onset, study medication allergy, and subsequent central vertigo diagnosis. Owing to ethical concerns and the possible adverse effects of sodium bicarbonate, the exclusion criteria included patients with New York Heart Association greater than stage 1 heart failure and greater than stage 2 chronic kidney disease.

### Intervention and Randomization

The trial was limited to the daytime on weekdays because of the research associates’ unavailability during nights and weekends. Patients with dizziness or vertigo were assessed by ED physicians to ensure they met the inclusion criteria. Medical history assessments and neurologic examinations were performed to exclude central vertigo and other conditions. Patients with a diagnosis of acute peripheral vertigo were invited by ED physicians to participate in the trial. Emergency department physicians activated a trained research associate if an eligible patient agreed to participate. Patients who declined were treated based on the physician’s clinical judgment and expertise. Patients with special and unstable conditions (eg, symptom resolution before randomization, low blood pressure, or unreachable research associates) were excluded from the randomization process.

Eligible patients who provided written informed consent were randomly assigned to 1 of 3 groups in a 1:1:1 ratio. The randomization process involved a block size of 9, where the random order was generated by a computer and sealed in envelopes before the trial began. After reconfirming patient eligibility and obtaining informed consent, the research associate handed over a sealed envelope containing a randomized assignment to the primary ED physician. The medical team prepared and administered the selected medications according to standard procedures while blinding the patients and research associates. The research associates were absent during drug administration, and the participants were not informed of the drug name.

The prescribed medication was administered within 10 to 20 minutes. Patients allocated to the standard treatment group (group A) received 30 mg of diphenhydramine (diphenhydramine hydrochloride injection 3%, 30 mg/1 mL/amp; Ying Yuan Chemical Pharmaceutical Corp) in 100 mL of normal saline solution administered via intravenous infusion. Patients allocated to the intervention groups were treated with sodium bicarbonate (group B) or combination therapy (group C). Patients in the sodium bicarbonate group (group B) received 66.4 mEq (approximately 1 mEq/kg) of sodium bicarbonate (Rolikan injection 7% sodium bicarbonate, 16.6 mEq/20 mL/amp; Taiwan Biotech Corp) in 100 mL of normal saline solution administered via intravenous infusion. To avoid the hypotensive effect of rapid diphenhydramine infusion and ensure that the medication was administered within 10 to 20 minutes, patients in the combination therapy group (group C) received 30 mg of diphenhydramine in 100 mL of normal saline solution intravenously and 66.4 mEq of sodium bicarbonate intravenously, slowly pushed for 2 minutes.

The research associate performed 5 evaluations across 3 visits for the study patients: at baseline before drug administration, 30 minutes after drug administration, and 3 evaluations at 60 minutes. Evaluation details are presented in the Data Collection subsection. Then, the ED physicians in charge discharged patients directly, provided rescue management, performed repositioning maneuvers, observed patients in the ED, or arranged further imaging examinations based on treatment response and physician expertise. Breakthrough interventions by ED physicians were permitted if the patient experienced unexpected critical changes during the allocated treatment.

### Data Collection

Patient vital signs and blood pressure data were obtained during triage. A standardized form comprising the patient’s medical history and vertigo-related information was completed after stabilization. Blood cell count and sodium, random blood glucose, and venous blood gas levels were measured before drug administration.

A research associate, certified and trained in accordance with the institutional review board regulations, collected data. A 2-week pretraining process was conducted, and the authors validated the data collection process. Vertigo and nausea severity were assessed using a 10-point visual analog scale (VAS) before drug administration and 30 and 60 minutes later.^9^ Patients were instructed by the research associate to indicate their sensation level by circling a single number on the scale, with 10 representing maximum sensation and 0 representing no sensation. Vertigo severity was also recorded using the VAS after head rotation and ambulation tests. Patients were asked to turn their head 45° to the right and then to the left and attempt ambulation while standing. Patients were also asked about their subjective perception of ambulation limitations, and their responses were categorized as normal, mild, moderate, or severe. This assessment was conducted before drug administration and 60 minutes after the ambulation test. The patients were asked about their subjective feelings of lethargy (categorized as normal, mild, moderate, or severe) and any injection pain when the trial ended. After the trial, the patients were followed up at the outpatient clinic or by telephone for 14 days or more to ensure that no major adverse events or hospital admissions occurred.

### Outcomes

The primary outcome was the change in vertigo VAS score from baseline to 60 minutes after drug administration. Secondary outcomes included the change in vertigo VAS scores from baseline to 30 minutes, change in nausea VAS scores from baseline to 30 and 60 minutes, change in vertigo VAS scores from sitting to head rotation and ambulation at 60 minutes, subjective feelings of the limitation of ambulation at 60 minutes, ED length of stay, and any further rescue management used by ED physicians after the trial. The ED length of stay was recorded as 24 hours if the patient was admitted or stayed in the ED for more than 24 hours.

### Sample Size Estimation

The target sample size was estimated based on the comparison of changes (difference in differences) in vertigo VAS scores from baseline to 60 minutes among the 3 treatment groups using a 3-way repeated-measures analysis of variance (ANOVA). We assumed a medium effect size of 0.25, an α error of .05, 90% power, and a 10% dropout rate. Based on these assumptions, we determined that 75 patients would be required for each arm of the trial. Sample size estimation was calculated using general power (G-Power) software, version 3.1.^24^

### Statistical Analysis

Analysis was performed on a modified intention-to-treat basis, with postrandomization exclusions applied to patients who met the protocol-specified criteria for central vertigo. Categorical variables are reported as numbers and percentages. For continuous variables, normal distribution was considered if skewness or kurtosis values fell between 2 and −2. Normally distributed data are reported as mean (SD) values, and nonnormally distributed data are reported as median (IQR) values. To compare the primary outcome, 3-way ANOVA was used to assess differences among the 3 groups. For secondary outcomes, the χ^2^ test was used for categorical variables, whereas 3-way ANOVA was used for normally distributed data and the Kruskal-Wallis test was used for nonnormally distributed data. For the subgroup analysis, we evaluated the treatment effect between group A and groups B and C among subgroups of age, sex, previous vertigo episode, and onset time. For the continuous variables of age and vertigo onset time, the median cutoff point was selected. When the overall test result was significant, post hoc tests were conducted with Bonferroni correction by multiplying the individual *P* values by 3 and comparing them with the .05 threshold.

In addition to the single-variable analysis, a generalized estimating equation (GEE) model with an unstructured correlation matrix was used to investigate the longitudinal relationship between the 3 treatment groups and vertigo VAS score changes over the 4 evaluation points across the survey. The coefficient associated with the interaction term between the grouping variable and the evaluation in the GEE model indicated how the effect of the grouping variable changes across different evaluations from the baseline. We also adjusted for potential confounding factors that may affect the vertigo VAS scores, including the categorical variable sex and the continuous variables age and vertigo onset time. Data were processed and analyzed using SPSS Statistics, version 26 (IBM Corp). All *P* values were from 2-sided tests, and results were deemed statistically significant at *P* < .05.

## Results

### Patient Recruitment and Characteristics

**Figure 1.**
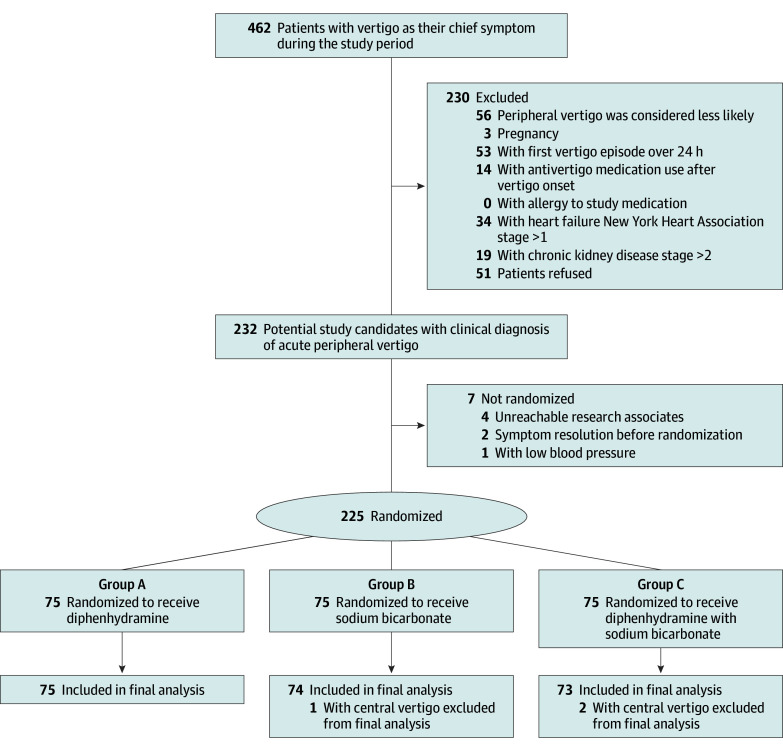
Flowchart of the Study Patients

During the study period, 462 patients with dizziness or vertigo underwent an eligibility assessment, of whom 225 participated in this study. Patients were randomly assigned in a 1:1:1 ratio to receive the intended treatment. The trial ended when the estimated sample size was reached. All participants were followed up for 14 days after the trial. Three participants (1 in group B and 2 in group C) were excluded from the final analysis because of a diagnosis of central vertigo after randomization (Figure 1). One patient in group B was admitted on day 3 because of gastrointestinal bleeding.

The final population of 222 patients included 150 women (67.6%) and 72 men (32.4%), with a mean (SD) age of 57.9 (17.6) years (Table 1). Approximately 59.9% of patients (n = 133) had experienced vertigo before, and the mean (SD) time from symptom onset to the ED visit was 6.5 (6.6) hours. The baseline characteristics of patients in each group are presented in Table 1. The intraclass correlation coefficient value for the baseline vertigo VAS score in this trial was 0.02.

**Table 1.** Baseline Characteristics of the Study Population

Characteristic	Group A (n = 75)^a^	Group B (n = 74)^a^	Group C (n = 73)^a^
Demographic information			
Age, mean (SD), y	59.2 (18.3)	53.7 (16.3)	61.0 (17.4)
Sex, No. (%)			
Male	21 (28.0)	27 (36.5)	24 (32.9)
Female	54 (72.0)	47 (63.5)	49 (67.1)
Activities of daily living, No. (%)			
Independent	71 (94.7)	71 (95.9)	68 (93.2)
Partial dependent	4 (5.3)	3 (4.1)	5 (6.8)
Comorbid conditions, No. (%)			
Diabetes	12 (16.0)	9 (12.2)	12 (16.4)
Hypertension	30 (40.0)	26 (35.1)	33 (45.2)
Coronary artery disease	8 (10.7)	5 (6.8)	8 (11.0)
Chronic obstructive lung disease	4 (5.3)	4 (5.4)	9 (12.3)
Cerebral vascular accident	2 (2.7)	1 (1.4)	4 (5.5)
Vertigo-related history			
With vertigo history, No. (%)	47 (62.7)	42 (56.8)	44 (60.3)
Time from symptoms onset, mean (SD), h	7.1 (7.3)	7.0 (7.0)	5.4 (5.2)
Symptoms at arrival, No. (%)			
Progression	25 (33.3)	14 (18.9)	19 (26.0)
Persistent	44 (58.7)	53 (71.6)	47 (64.4)
Improving	6 (8.0)	7 (9.5)	7 (9.6)
Difficulty in eye opening	29 (38.7)	27 (36.5)	23 (31.5)
Nausea	62 (82.7)	58 (78.4)	56 (76.7)
Ambulatory limitation, No. (%)			
Normal	15 (20.3)	15 (20.8)	13 (18.1)
Mild	24 (32.4)	27 (37.5)	27 (37.5)
Moderate	28 (37.8)	26 (36.1)	26 (36.1)
Severe	7 (9.5)	4 (5.6)	6 (8.3)
Examination results before drug administration			
Triage HR, mean (SD), beats/min	77.6 (15.5)	77.7 (14.3)	81.5 (15.4)
Triage BP, mean (SD), mm Hg	101.9 (14.3)	102.6 (16.1)	106.9 (15.0)
WBC count, mean (SD), cells/µL	7720 (2300)	7870 (2520)	7630 (2690)
Hemoglobin, mean (SD), g/dL	13.2 (1.8)	13.6 (2.0)	13.4 (1.6)
Sodium, mean (SD), mEq/L	137.3 (2.3)	136.9 (2.4)	136.3 (3.3)
pH, mean (SD)	7.385 (0.046)	7.386 (0.045)	7.391 (0.051)
CO_2_, mean (SD), mm Hg	41.6 (7.8)	42.4 (6.9)	41.7 (5.9)
Bicarbonate, mean (SD), mEq/L	24.4 (2.8)	24.7 (3.2)	24.6 (1.8)

Abbreviations: BP, blood pressure; HR, heart rate; WBC, white blood cell.

SI conversion factors: To convert WBC count to ×10^9^ per liter, multiply by 0.001; hemoglobin to grams per liter, multiply by 10.0; sodium to millimoles per liter, multiply by 1.0; and bicarbonate to millimoles per liter, multiply by 1.0.

^a^ Group A received 30 mg of diphenhydramine in 100 mL of normal saline solution administered via intravenous infusion, group B received 66.4 mEq (approximately 1 mEq/kg) of sodium bicarbonate in 100 mL of normal saline solution administered via intravenous infusion, and group C received 30 mg of diphenhydramine in 100 mL of normal saline solution intravenously and 66.4 mEq of sodium bicarbonate intravenously, slowly pushed for 2 minutes.

### Primary and Secondary Outcomes

**Figure 2.**
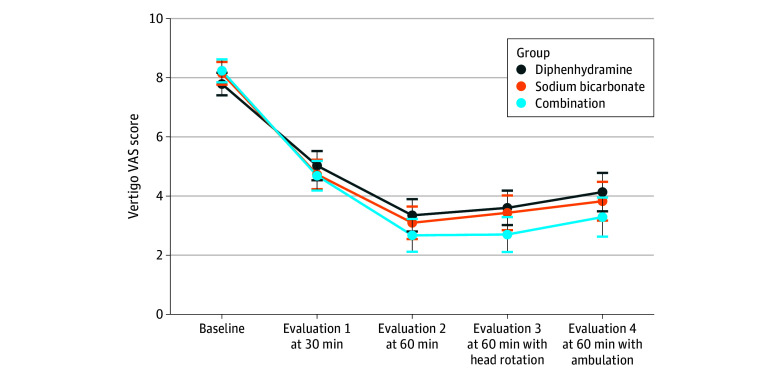
Vertigo Visual Analog Scale (VAS) Score at Baseline and Each Evaluation Error bars indicate 95% CIs.

The baseline mean (SD) vertigo VAS scores were 7.8 (1.9) in group A, 8.2 (1.6) in group B, and 8.2 (1.5) in group C. The changes in vertigo VAS score in each group after medical treatment are shown in Figure 2.

Regarding the primary outcome, the mean (SD) changes in vertigo VAS score from baseline to 60 minutes were −4.4 (2.7) in group A, −5.1 (2.2) in group B, and −5.6 (2.1) in group C (Table 2). These changes were statistically significant in the ANOVA analysis (*P* = .02). Post hoc analysis showed a better outcome in group C than in group A (*P* = .01).

**Table 2.** Treatment Outcomes and Adverse Effects

Outcome	Group A (n = 75)^a^	Group B (n = 74)^a^	Group C (n = 73)^a^	*P* value	*P* value^b^		
					A vs B	A vs C	B vs C
Primary outcome: postmedication vertigo sensation change at 60 min, mean (SD)							
Baseline vertigo VAS score	7.8 (1.9)	8.2 (1.6)	8.2 (1.5)	NA	NA	NA	NA
Vertigo VAS score change at 60 min	−4.4 (2.7)	−5.1 (2.2)	−5.6 (2.1)	.02^c^	.34	.01^c^	.57
Secondary outcomes: postmedication vertigo sensation change at 30 min, mean (SD)							
Vertigo VAS score change at 30 min	−2.8 (2.1)	−3.4 (1.9)	−3.6 (2.1)	.04^c^	.14	.05	>.99
Postmedication nausea sensation change, mean (SD)							
Baseline nausea VAS score	5.6 (3.5)	5.6 (3.7)	6.0 (3.8)	NA	NA	NA	NA
Nausea VAS score change at 30 min	−3.3 (3.0)	−2.4 (2.3)	−3.8 (3.3)	.02^c^	.25	.78	.01^c^
Nausea VAS score change at 60 min	−4.3 (3.3)	−4.2 (3.4)	−5.3 (3.6)	.10	NA	NA	NA
Alteration in vertigo sensation after movement, mean (SD)							
Vertigo VAS score at 60 min	3.4 (2.6)	3.1 (2.4)	2.7 (2.2)	NA	NA	NA	NA
Vertigo VAS score change during head rotation	0.3 (1.1)	0.3 (1.2)	0.03 (0.8)	.17	NA	NA	NA
Vertigo VAS score change during ambulation	0.8 (1.2)	0.7 (1.5)	0.6 (1.0)	.71	NA	NA	NA
Ambulatory limitation at 60 min, No. (%)							
Normal	22 (29.7)	33 (45.8)	34 (47.2)	.28	NA	NA	NA
Mild	35 (47.3)	29 (40.3)	28 (38.9)				
Moderate	10 (13.5)	4 (5.6)	6 (8.3)				
Severe	7 (9.5)	6 (8.3)	4 (5.6)				
Emergency department length of stay, median (IQR), h	2.3 (1.3)	2.1 (1.5)	2.0 (1.2)	.03^c^	.07	.07	>.99
Need for rescue management, No. (%)							
No	40 (53.3)	49 (66.2)	60 (82.2)	.001^c^	.33	<.001^c^	.08
Yes	35 (46.7)	25 (33.8)	13 (17.8)				
Lethargy, No. (%)							
No	16 (21.3)	51 (68.9)	20 (27.4)	.001^c^	.001^c^	.46	<.001^c^
Mild	29 (38.7)	15 (20.3)	25 (34.2)				
Moderate	29 (38.7)	6 (8.1)	22 (30.1)				
Severe	1 (1.3)	2 (2.7)	6 (8.2)				
Injection discomfort, No. (%)							
No	69 (92.0)	61 (82.4)	52 (71.2)	.004^c^	.24	.003^c^	.32
Yes	6 (8.0)	13 (17.6)	21 (28.8)				

Abbreviations: NA, not applicable; VAS, visual analog scale.

^a^ Group A received 30 mg of diphenhydramine in 100 mL of normal saline solution administered via intravenous infusion, group B received 66.4 mEq (approximately 1 mEq/kg) of sodium bicarbonate in 100 mL of normal saline solution administered via intravenous infusion, and group C received 30 mg of diphenhydramine in 100 mL of normal saline solution intravenously and 66.4 mEq of sodium bicarbonate intravenously, slowly pushed for 2 minutes.

^b^ With Bonferroni correction.

^c^ Significant at *P* < .05.

Regarding secondary outcomes, the mean (SD) change in nausea VAS score from baseline to 30 minutes showed a better outcome in group C than in group B (–3.8 [3.3] vs –2.4 [2.3]; *P* = .01) (Table 2). There were no significant differences in the 30th minute of vertigo and 60th minute of nausea VAS score change, alteration in vertigo sensation after movement, subjective feelings of the limitations of ambulation after treatment, and ED stay time among the groups. Rescue therapy was used less frequently in group C (17.8% [13 of 73]) than in group A (46.7% [35 of 75]; *P* < .001).

More than 70% of patients who received diphenhydramine (59 of 75 patients in group A [78.7%] and 53 of 73 patients in group C [72.6%]) experienced lethargy after administration (Table 2). Groups treated with diphenhydramine had a greater chance of experiencing moderate lethargy (group A, 38.7% [29 of 75]; group B, 8.1% [6 of 74]; group C, 30.1% [22 of 73]; *P* < .001), whereas groups treated with sodium bicarbonate had a greater chance of experiencing injection discomfort (group A, 8.0% [6 of 75]; group B, 17.6% [13 of 74]; group C, 28.8% [21 of 73]; *P* = .004). No other severe adverse events, such as drug allergies or hypotension, were recorded during the trial.

### Subgroup Analysis

The better treatment effect in group C vs group A persisted among male patients (mean [SD] change in vertigo VAS score, –5.3 [1.7] vs –3.4 [2.6]; *P* = .003), younger patients (mean [SD] change in vertigo VAS score, –5.8 [1.9] vs –3.8 [3.2]; *P* = .006), and patients without previous vertigo episodes (mean [SD] change in vertigo VAS score, –6.2 [1.6] vs –4.4 [3.0]; *P* = .008) (Table 3). A better treatment effect was found in group B than in group A among male patients (mean [SD] change in vertigo VAS score, –5.5 [1.3] vs –3.4 [2.6]; *P* = .001) and patients without previous vertigo episodes (mean [SD] change in vertigo VAS score, –5.9 [1.8] vs –4.4 [3.0]; *P* = .03).

**Table 3.** Subgroup Analysis Results of Postmedication Change in Vertigo VAS Score at 60 Minutes

Characteristic	No.	Change in vertigo VAS score, mean (SD)			*P* value	*P* value^a^		
		Group A^b^	Group B^b^	Group C^b^		A vs B	A vs C	B vs C
Sex								
Male	72	−3.4 (2.6)	−5.5 (1.3)	−5.3 (1.7)	<.001^c^	.001^c^	.003^c^	>.99
Female	150	−4.9 (2.6)	−4.8 (2.6)	−5.7 (2.3)	.14	NA	NA	NA
Age, y								
>60	111	−5.1 (1.9)	−5.1 (2.2)	−5.4 (2.2)	.74	NA	NA	NA
≤60	111	−3.8 (3.2)	−5.0 (2.3)	−5.8 (1.9)	.01^c^	.12	.006^c^	.60
Onset time, h								
>4	93	−4.5 (2.6)	−4.6 (2.1)	−5.5 (2.0)	.18	NA	NA	NA
≤4	129	−4.4 (2.8)	−5.3 (2.8)	−5.6 (2.2)	.05	NA	NA	NA
With vertigo history								
Yes	133	−4.5 (2.5)	−4.4 (2.3)	−5.2 (2.3)	.28	NA	NA	NA
No	89	−4.4 (3.0)	−5.9 (1.8)	−6.2 (1.6)	.006^c^	.03^c^	.008^c^	1.00

Abbreviations: NA, not applicable; VAS, visual analog scale.

^a^ With Bonferroni correction.

^b^ Group A received 30 mg of diphenhydramine in 100 mL of normal saline solution administered via intravenous infusion, group B received 66.4 mEq (approximately 1 mEq/kg) of sodium bicarbonate in 100 mL of normal saline solution administered via intravenous infusion, and group C received 30 mg of diphenhydramine in 100 mL of normal saline solution intravenously and 66.4 mEq of sodium bicarbonate intravenously, slowly pushed for 2 minutes.

^c^ Significant at *P* < .05.

### Ancillary Analyses

In a GEE logistic model including the interaction between treatment group and repeated measurement, baseline VAS score differences between the groups were not statistically significant (eTable in Supplement 2). Sex, age, and onset time were not significantly associated with the VAS score. When considering VAS scores in each evaluation, VAS scores decreased significantly at all subsequent evaluations compared with those at baseline. Significant interactions between treatment groups and repeated measurements were observed, indicating that the longitudinal pattern of treatment responses in groups B and C were different from group A. For group C, significant reductions in VAS scores relative to group A were observed in 4 evaluations, whereas for group B, a significant reduction in VAS scores relative to group A was observed only at evaluation 1.

## Discussion

In this double-blinded, randomized clinical trial, although diphenhydramine was the standard treatment for acute peripheral vertigo, first, we found that the single use of either diphenhydramine or sodium bicarbonate effectively reduced the VAS scores. Second, combining diphenhydramine with sodium bicarbonate further reduced vertigo symptoms compared with diphenhydramine alone. Third, the positive effect of combination therapy was significantly predominant among male patients, younger individuals, and those without vertigo history compared with diphenhydramine alone.

### Comparison With Current Evidence

The efficacy of sodium bicarbonate in treating vertigo is rarely discussed in Western countries, although it is widely used in Eastern countries (eg, Taiwan and Japan). Although positive effects have been reported in the Chinese literature,^18^ the current evidence remains limited to English language–based studies.^19,25^ To date, no other trials have investigated sodium bicarbonate for vertigo treatment, according to the International Clinical Trials Registry Platform. To our knowledge, this is the first double-blinded, randomized clinical trial to demonstrate that sodium bicarbonate has a similar effect compared with diphenhydramine in treating acute peripheral vertigo.

### Possible Mechanism

In animal models, sodium bicarbonate directly inhibits neural activity in the medial vestibular nucleus.^21^ In a recent animal study, sodium bicarbonate reduced amygdala activation after exposure to hyper gravity.^22^ Furthermore, sodium bicarbonate increased cochlear blood flow in normal or endolymphatic hydrops in an animal study.^23^ In a human study, increased cerebral blood flow was observed after sodium bicarbonate infusion during isocapnic breathing.^20^ Nevertheless, these findings do not fully explain the additional benefits of sodium bicarbonate among male patients and younger patients observed in this study.

### Clinical Application

Because our trial reported that more than 70% of patients experienced lethargy after diphenhydramine administration, sodium bicarbonate may be a safer vertigo treatment alternative owing to its lower risk of lethargic effects. This is particularly important for patients who need to drive or work, as well as for ED patients who are at a higher risk of falls. Patients treated with sodium bicarbonate should be informed of possible tenderness during and after injection or of the subsequent risk of vasculitis; slow infusion administration may slightly reduce this risk. Based on our subgroup analysis, younger male patients without a history of vertigo showed reduced response to diphenhydramine treatment. Sodium bicarbonate or combination therapy can be considered for these patient groups.

### Limitations

This study has some limitations. First, our study was conducted exclusively during the daytime because of limited staff resources. However, there is no evidence to suggest that vertigo treatment is affected by circadian rhythms. Second, to avoid interference with ED workflows, medical staff were not blinded to the randomization process. Some secondary outcomes related to physicians’ decisions, including rescue management needs and ED length of stay, should be interpreted with caution. Third, the primary outcome was based on patients’ subjective perception of vertigo on the VAS. No objective surrogate primary outcome was used in this study. Although there was no accepted minimal clinically significant difference in vertigo VAS scores in the originally developed study,^9^ VAS became a standardized, validated reporting format to evaluate the effects of the treatment in subsequent studies.^12,13,14^ Future studies need to focus on the clinical utility of the VAS. Fourth, our study lacked a placebo arm, so we cannot determine the isolated effects of diphenhydramine, sodium bicarbonate, or their combination compared with a placebo. The treatment effect of reducing the VAS score in this study referred to the change from baseline. However, given that antihistamines are regarded as standard therapy in emergency medicine practice and textbooks,^26^ we consider the absence of a placebo arm ethically reasonable. Fifth, the specific type of peripheral vertigo could not be determined in this study. Thus, further studies should focus on identifying which subtypes of peripheral vertigo may benefit from sodium bicarbonate treatment. Sixth, the number of patients was insufficient to provide adequate power for subgroup analyses. Seventh, the findings’ generalizability must be validated through multicenter studies.

## Conclusions

The findings of this randomized clinical trial indicate that combination therapy with diphenhydramine and sodium bicarbonate can further reduce vertigo sensation compared with diphenhydramine alone, especially among younger male patients without a vertigo history. Further studies should focus on the determination of peripheral vertigo types that can benefit from sodium bicarbonate and the underlying mechanisms.

## Notes

Supplement 1.
Trial Protocol and Statistical Analysis Plan

Supplement 2. **eTable.** GEE Model: Temporal and Interactive Effects of the Treatment Group

Supplement 3.
Data Sharing Statement
