# Error in Visual Abstract

**DOI:** 10.1001/jamanetworkopen.2025.54180

**Published:** 2025-12-29

In the Original Investigation titled “Diphenhydramine, Sodium Bicarbonate, or Combination for Acute Peripheral Vertigo: A Randomized Clinical Trial”^1^ published online on November 6, 2025, there was an error in the Intervention section of the Visual Abstract. For the 75 patients who were randomized to receive a combination of diphenhydramine and sodium bicarbonate, the dose of diphenhydramine was 30 mg, not 3 mg. This article was corrected.^1^
