# Supplementary material for: Diphenhydramine, Sodium Bicarbonate, or Combination for Acute Peripheral Vertigo: A Randomized Clinical Trial
Source: JAMA Netw Open. 2025 Nov 6;8(11):e2541472. doi: 10.1001/jamanetworkopen.2025.41472 (PMC12593102; doi:10.1001/jamanetworkopen.2025.41472)
Supplement: Supplement 1. — Trial Protocol and Statistical Analysis Plan [file jamanetwopen-e2541472-s001.pdf]

Clinical Trial Protocol

Comparison between the Efficacy of Diphenhydramine, Sodium Bicarbonate, and Their Combination in Treating Acute Peripheral Vertigo: A Double-Blinded Randomized Clinical Trial

Short Title: Diphenhydramine, Sodium Bicarbonate, and Their Combination for Vertigo

Study Site: Emergency Department, National Taiwan University Hospital Yunlin Branch

Principal Investigator: Chien-Yu Chi

Sponsor: National Taiwan University Hospital

Planned Study Period: 01/01/2023 to 12/31/2023

Protocol Version: Version 4.0

IRB No.: NTUH-REC No. 202209092MIND

Table of Contents

1. Acronyms and Abbreviations 3

2. Trial Summary 4

3. Background 4

4. Trial Design 4

4.1. Hypothesis 4

4.2. Inclusion Criteria 4

4.3. Exclusion Criteria 4

4.4. Trial Setting 4

4.5. Randomization and Blinding 5

4.6. Intervention 5

5. Outcomes Measurement 5

5.1. Primary Outcome 5

5.2. Secondary Outcomes 5

6. Sample Size Estimation 5

7. Data Collection 5

8. Statistical Analysis 5

9. Interim analysis and stopping criteria 6

10. Reference 6

1. Acronyms and Abbreviations

ED	Emergency Department
VAS	Visual Analog Scale
NYHA	New York Heart Association
CKD	Chronic Kidney Disease
GEE	Generalized Estimating Equations

2. Trial Summary

This randomized, double-blinded clinical trial evaluates the efficacy of sodium bicarbonate, diphenhydramine, and their combination in patients presenting to the emergency department (ED) with acute peripheral vertigo. Conducted at the National Taiwan University Hospital Yunlin Branch's ED. The trial aims to provide evidence-based recommendations for acute vertigo management by assessing symptom relief, safety profiles, and potential improvements in patient outcomes.

3. Background

Vertigo is a common ED complaint characterized by spinning sensations and severe nausea. Approximately 3.13% of adults in Taiwan experience vertigo annually, with a high recurrence rate. Antihistamines like diphenhydramine are standard treatments but often cause sedation and increased fall risk. Sodium bicarbonate, traditionally used for metabolic conditions, offers an alternative without sedative effects. However, systematic comparative research is lacking, necessitating this trial.

4. Trial Design

This study is a triple-arm, double-blinded, randomized clinical trial comparing diphenhydramine, sodium bicarbonate, and their combination. The trial will enroll adult patients with acute peripheral vertigo presenting within 24 hours of symptom onset.

4.1. Hypothesis

This study aimed to evaluate the efficacy of sodium bicarbonate, both alone and in combination with an antihistamine, compared to antihistamine monotherapy in treating acute peripheral vertigo in ED patients. We hypothesized that the treatments would demonstrate varying levels of effectiveness due to their distinct mechanisms of action.

4.2. Inclusion Criteria

Adults aged 18 years or older presenting with peripheral vertigo within 24 hours of onset.

4.3. Exclusion Criteria

Pregnancy, prior anti-vertigo medication use after vergio onset, heart failure New York Heart Association (NYHA) class >1, chronic kidney disease (CKD) stage >2, allergy to study drugs, and central vertigo diagnosis.

4.4. Trial Setting

The trial is conducted at the ED of the National Taiwan University Hospital Yunlin Branch, which serves approximately 300,000 people and has 1,000 beds, and receives approximately 3,500 ED visits each month.

4.5. Randomization and Blinding

The randomization process used a block size of 9, with the allocation sequence computer-generated and sealed in envelopes prior to trial initiation. This approach ensured that both patients and the research associate remained blinded to treatment assignments.

4.6. Intervention

Group A receives diphenhydramine 30 mg intravenous dripping in 100 mL saline; Group B receives sodium bicarbonate 66.4 mEq intravenous dripping in 100 mL saline; Group C receives combination therapy of diphenhydramine 30 mg intravenous dripping in 100 mL saline and sodium bicarbonate 66.4 mEq intravenous via slow push.

5. Outcomes Measurement

5.1. Primary Outcome

Change in vertigo severity measured by the Visual Analog Scale (VAS) from baseline to 60 minutes post-treatment.

5.2. Secondary Outcomes

Changes in vertigo and nausea VAS scores at 30 and 60 minutes, changes in vertigo VAS scores after head-turn and ambulation tests, subjective ambulation limitations, ED stay duration, and need for additional rescue treatments post-trial.

6. Sample Size Estimation

Sample size was calculated based on a three-way repeated-measures ANOVA, with a medium effect size (0.25), alpha of 0.05, 90% power, and 10% dropout rate, resulting in a requirement of 75 patients per group (225 total).

7. Data Collection

Vertigo and nausea severity were assessed using a 10-point VAS before treatment and at 30- and 60-minutes post-administration. Additional evaluations included VAS scores after head-turn and ambulation tests, and subjective ratings of ambulation difficulty and lethargy (categorized as normal to severe). Injection site pain was also recorded. Patients were followed up for at least 14 days post-trial to monitor for major adverse events or hospitalizations.

8. Statistical Analysis

The statistical analysis will involve evaluating the primary outcome, which is the reduction in vertigo VAS scores between treatment groups, using ANOVA or Kruskal–Wallis tests depending on data normality. Secondary outcomes will be analyzed similarly, with necessary adjustments for multiple comparisons using appropriate post-hoc tests. Subgroup analyses will be performed to explore variations in treatment effects based on age, sex, previous vertigo episodes, and symptom onset time. A generalized estimating equation (GEE) model with an unstructured correlation

matrix was employed to examine the longitudinal association between the three treatment groups and changes in vertigo VAS scores across time points.

9. Interim analysis and stopping criteria

Interim analysis and stopping criteria were not applicable in this study due to its relatively simple design and short study duration. Moreover, the medications used—sodium bicarbonate and diphenhydramine—have well-established safety profiles and are commonly used in clinical practice.

10. Reference

1. Neuhauser HK. Chapter 5 - The epidemiology of dizziness and vertigo. In: Furman JM, Lempert T, eds. Handbook of Clinical Neurology: Elsevier; 2016:67-82.
2. Neuhauser HK, Radtke A, von Brevern M, Lezius F, Feldmann M, Lempert T. Burden of dizziness and vertigo in the community. Arch Intern Med 2008;168:2118-24.
3. Lai YT, Wang TC, Chuang LJ, Chen MH, Wang PC. Epidemiology of vertigo: a National Survey. Otolaryngol Head Neck Surg 2011;145:110-6.
4. Baloh RW. Differentiating between peripheral and central causes of vertigo. Otolaryngol Head Neck Surg 1998;119:55-9.
5. Kerber KA, Brown DL, Lisabeth LD, Smith MA, Morgenstern LB. Stroke among patients with dizziness, vertigo, and imbalance in the emergency department: a population-based study. Stroke 2006;37:2484-7.
6. Casani AP, Gufoni M, Capobianco S. Current Insights into Treating Vertigo in Older Adults. Drugs Aging 2021;38:655-70.
7. Strupp M, Arbusow V, Brandt T. Exercise and drug therapy alter recovery from labyrinth lesion in humans. Ann N Y Acad Sci 2001;942:79-94.
8. Bhattacharyya N, Gubbels SP, Schwartz SR, Edlow JA, El-Kashlan H, Fife T, et al. Clinical Practice Guideline: Benign Paroxysmal Positional Vertigo (Update). Otolaryngology-Head and Neck Surgery 2017;156:S1-S47.
9. Shih RD, Walsh B, Eskin B, Allegra J, Fiesseler FW, Salo D, et al. Diazepam and Meclizine Are Equally Effective in the Treatment of Vertigo: An Emergency Department Randomized Double-Blind Placebo-Controlled Trial. J Emerg Med 2017;52:23-7.
10. Ozdemir H, Akinci E, Coskun F. Comparison of the effectiveness of intravenous piracetam and intravenous dimenhydrinate in the treatment of acute peripheral vertigo in the emergency department. Singapore Med J 2013;54:649-52.
11. Marill KA, Walsh MJ, Nelson BK. Intravenous Lorazepam versus dimenhydrinate for treatment of vertigo in the emergency department: a randomized clinical trial. Ann Emerg Med 2000;36:310-9.
12. Irving C, Richman P, Kaiafas C, Eskin B, Allegra J. Intramuscular droperidol versus intramuscular dimenhydrinate for the treatment of acute peripheral vertigo in the emergency department: a randomized clinical trial. Acad Emerg Med 2002;9:650-3.
13. Ercin D, Erdur B, Turkcuer I, Seyit M, Ozen M, Yilmaz A, et al. Comparison of efficacy dimenhydrinate and metoclopramide in the treatment of nausea due to vertigo; a randomized study. Am J Emerg Med 2021;40:77-82.
14. Doğan N, Avcu N, Yaka E, Yılmaz S, Pekdemir M. Comparison of the therapeutic efficacy of

intravenous dimenhydrinate and intravenous piracetam in patients with vertigo: a randomised clinical trial. Emerg Med J 2015;32:520-4.
15. Amini A, Heidari K, Asadollahi S, Habibi T, Shahrami A, Mansouri B, et al. Intravenous promethazine versus lorazepam for the treatment of peripheral vertigo in the emergency department: A double blind, randomized clinical trial of efficacy and safety. J Vestib Res 2014;24:39-47.
16. Hunter BR, Wang AZ, Bucca AW, Musey PI, Jr., Strachan CC, Roumpf SK, et al. Efficacy of Benzodiazepines or Antihistamines for Patients With Acute Vertigo: A Systematic Review and Meta-analysis. JAMA Neurol 2022.
17. Winblad B. Piracetam: a review of pharmacological properties and clinical uses. CNS Drug Rev 2005;11:169-82.
18. Cheng YC, Lee WC, Kuo LC, Lin YK, Chen CW, Lin TY, et al. Use of sodium bicarbonate for acute dizziness after minor head injury. Tzu Chi Medical Journal 2011;23:46-50.
19. Kawabata A, Kishimoto T, Ujihara H, Sasa M, Takaori S. Inhibition by intravenously administered sodium bicarbonate of neuronal activity in medial vestibular nucleus neurons. Japanese Journal of Pharmacology 1990;54:383-9.
20. Numata K, Shiga T, Omura K, Umibe A, Hiraoka E, Yamanaka S, et al. Comparison of acute vertigo diagnosis and treatment practices between otolaryngologists and non-otolaryngologists: A multicenter scenario-based survey. PLoS One 2019;14:e0213196.
21. S-Y. C, Y-H. Y. Sodium Bicarbonate for the Therapeutic Diagnosis of Acute Vertiginous Attack. The Journal of Taiwan Otolaryngology-Head and Neck Surgery 1995;30:418-22.
