# Supplementary material for: Diphenhydramine, Sodium Bicarbonate, or Combination for Acute Peripheral Vertigo: A Randomized Clinical Trial
Source: JAMA Netw Open. 2025 Nov 6;8(11):e2541472. doi: 10.1001/jamanetworkopen.2025.41472 (PMC12593102; doi:10.1001/jamanetworkopen.2025.41472)
Supplement: Supplement 2. — eTable. GEE Model: Temporal and Interactive Effects of the Treatment Group [file jamanetwopen-e2541472-s002.pdf]

Supplementary Online Content

Chi CY, Chen YC, Cheng MT, et al. Diphenhydramine, sodium bicarbonate, or combination for acute peripheral vertigo: a randomized clinical trial. *JAMA Netw Open.* 2025;8(11):e2541472. doi:10.1001/jamanetworkopen.2025.41472

eTable. GEE Model: Temporal and Interactive Effects of the Treatment Group

This supplementary material has been provided by the authors to give readers additional information about their work.

© 2025 Chi CY et al. *JAMA Network Open.*

eTable. GEE Model: Temporal and Interactive Effects of the Treatment Group

**Factors**	**β**	Odds [95% CI]	P-value
**Main effect**			
Treatment group:			
Group A	Ref.		
Group B	0.35	1.41 [0.79 – 2.51]	.20
Group C	0.47	1.60 [0.91 – 2.82]	.10
Age, year	-0.006	0.99 [0.98 – 1.01]	.49
Onset time (h)	0.007	1.01 [0.96 – 1.06]	.80
Sex			
Female	Ref.		
Male	-0.40	0.67 [0.35 – 1.30]	.24
Time effect:			
Baseline vertigo VAS	Ref.		
Evaluation 1	-2.78	0.06 [0.04 – 0.10]	<.001 *
Evaluation 2	-4.46	0.01 [0.006 – 0.02]	<.001 *
Evaluation 3	-4.20	0.02 [0.008 – 0.03]	<.001 *
Evaluation 4	-3.67	0.03 [0.01 – 0.05]	<.001 *
**Interaction effect (treatment group x time)**			
Group B x Evaluation 1	-0.64	0.53 [0.28 – 0.99]	.05 *
Group B x Evaluation 2	-0.60	0.55 [0.25 – 1.21]	.14
Group B x Evaluation 3	-0.51	0.60 [0.26 – 1.40]	.24
Group B x Evaluation 4	-0.65	0.52 [0.21 – 1.31]	.16
Group C x Evaluation 1	-0.77	0.46 [0.24 – 0.90]	.02 *
Group C x Evaluation 2	-0.11	0.33 [0.15 – 0.71]	.005 *
Group C x Evaluation 3	-1.33	0.26 [0.12 – 0.58]	.001 *

© 2025 Chi CY et al. *JAMA Network Open.*

Group C x Evaluation 4	-1.28	0.28 [0.12 – 0.66]	.004 *

VAS, visual analog scale;

Evaluation 1, Vertigo VAS at 30min;

Evaluation 2, Vertigo VAS at 60min;

Evaluation 3, Vertigo VAS at 60min with head rotation;

Evaluation 4, Vertigo VAS at 60min with ambulation

© 2025 Chi CY et al. *JAMA Network Open.*
