# Supplementary material for: Diphenhydramine, Sodium Bicarbonate, or Combination for Acute Peripheral Vertigo: A Randomized Clinical Trial
Source: JAMA Netw Open. 2025 Nov 6;8(11):e2541472. doi: 10.1001/jamanetworkopen.2025.41472 (PMC12593102; doi:10.1001/jamanetworkopen.2025.41472)
Supplement: Supplement 3. — Data Sharing Statement [file jamanetwopen-e2541472-s003.pdf]

Data Sharing Statement

Chi. Diphenhydramine, Sodium Bicarbonate, or Combination for Acute Peripheral Vertigo. *JAMA Netw Open*. Published November 06, 2025. doi:10.1001/jamanetworkopen.2025.41472

Data
Additional Information: ClinicalTrials.gov Identifier: NCT05676216
Data available: Yes
Data types: Deidentified participant data
How to access data: Deidentified participant data can be accessed through the contact with the corresponding authors.
When available: With publication

Supporting Documents
Document types: None

Additional Information
Who can access the data: researchers whose proposed use of the data has been approved
Types of analyses: for research purposes with permission
Mechanisms of data availability: with investigator support
